# An online tomographic sediment trap for high-resolution environmental monitoring

**DOI:** 10.1007/s10661-026-15469-w

**Published:** 2026-05-22

**Authors:** Markus Johansson, Saija Saarni, Jouni Sorvari

**Affiliations:** 1 Department of Technical Physics, University of Eastern Finland, Kuopio, Finland; 2 Department of Geography and Geology, University of Turku, Turku, Finland; 3 Natural Resources Institute Finland (Luke), Helsinki, Finland

**Keywords:** Suspended sediment trapping, Online technology, Environmental monitoring, Flux rate, Aquatic sediments, Construction work impact, Water quality

## Abstract

Sediment trapping is a well-established method for obtaining information on sedimentation flux and composition in aquatic environments. Passive sediment traps typically have a collection period of several months to a year, while autonomous traps with revolving wheels can achieve a daily time resolution. However, these devices are often costly, require laborious maintenance, and have high power consumption, which limits their use in remote field locations. This study presents a novel, ultra-high-resolution (hourly temporal resolution) sediment trap based on optical tomography that enables continuous online monitoring of sediment flux. Laboratory validation demonstrated a high volumetric calibration accuracy, with a mean measurement error of ± 0.6 mL (*R*^2^ = 0.99). We deployed a prototype in Savilahti Bay, Kuopio, to evaluate its ability to detect short-term sediment pulses caused by onshore construction work. The field results confirmed the method’s effectiveness and reliability in tracking highly dynamic flux variations, although image processing techniques require further development for extreme sedimentation rates. The autonomous, solar-powered system transmits data wirelessly, significantly reducing fieldwork requirements. This methodology provides a crucial tool for monitoring high-resolution sediment dynamics and remote study sites.

**Supplementary Information:**

The online version contains supplementary material available at 10.1007/s10661-026-15469-w.

## Introduction

Suspended sediment trapping is a classic tool used to monitor sedimentation rates and the temporal variation in sediment composition in aquatic environments (Dymond et al., 1981; Liu et al., 2018; Ojala et al., 2013; Stockhecke et al., 2012). Sediment trapping became common in the late 1970 s, with extensive trap design and testing for various trap types being carried out (Dymond et al., 1981). Since then, sediment trapping has been used in environmental monitoring to measure the transport and sinking of environmental pollutants such as PAH compounds (Bellucci et al., 2016; Bouloubassi et al., 2006; Broman et al., 1988; Burniston et al., 2016), dioxins (Broman et al., 1989), heavy metals (Broman et al., 1988), and microplastics (Enders et al., 2019; Reineccius et al., 2020; Saarni et al., 2023) and to monitor attenuation processes in contaminated sites (Burniston et al., 2016; Lin et al., 2017). Trapping has been frequently used to better understand the transport and accumulation of a large variety of sedimentary components of allochthonous and autochthonous origin in aquatic environments (Kienel et al., 2017; Ojala et al., 2013, 2014; Pospelova et al., 2018; Price & Pospelova, 2011; Salmela et al., 2022) and to investigate processes controlling sediment influx (Bonk et al., 2015; Ojala et al., 2013; Stockhecke et al., 2012; Tylmann et al., 2012). Seasonal trapping has shed light on climatic controls on the lake systems (Bonk et al., 2015; Dean et al., 2015; Ojala et al., 2013; Tylmann et al., 2012), as well as the sediment dynamics in the 1000-km-long shelf systems (Liu et al., 2018).

Advances in sediment trapping include the collection of time series data on varying sedimentation rates and components, typically ranging from seasonal to even weekly resolution. However, trap maintenance is rather laborious especially in the remote sites and field work is costly, even more so, if short sampling time interval is desired. The automatic sediment traps today allow usually up to 12 sample bottles with pre-scheduled sampling period after which sample bottle is automatically changes. Yet temporal resolution of 2 weeks to one month is generally utilized to reduce field work interval. The automatic trap systems are often heavy and requires winch to be safely operated. Yet the need for the high-resolution time series of sediment transport and accumulation remains. Efficient ways in monitoring catchment erosion, sediment transport and accumulation are required to better understand effects of human land use, cultivation, industry, and infrastructural building as well as impacts of short term extreme weather events. Anthropogenic activities and land use changes are related to increase in sediment loading up to ten-fold (Dearing & Jones, 2003). Excess loading of sediments is significant and widespread form of aquatic pollution (Donohue & Molinos, 2009; Osterkamp et al., 1998) and can be a threat to ecological health of fresh waters through negative implications for the diversity, abundance and productivity of the biotic assemblages of water bodies (Donohue & Molinos, 2009) but also via changing light climate (Jeppesen et al., 1990), eutrophication (Tammelin & Kauppila, 2018) , and salinization (Kaushal et al., 2018). Restoration and protection management plans are only effective if sediment sources are identified. Furthermore, a shortage of monitoring data diminishes the meaningful control policies (Osterkamp et al., 1998). Large efforts for stabilizing cultivated areas to prevent soil and nutrient leaching are going on, and for monitoring the effectivity before and after stabilizing activities, an ultra-high resolution sediment trap could provide valuable data. Furthermore, to better understand sediment mobilization through extreme weather events at the changing climate in increasingly manmade environment ultra-high time resolution is required.

While there is a need for high-resolution environmental monitoring, resource limitations highlight the demand for autonomous sediment trapping instruments. Recent advancements in energy-efficient electronic components have made continuous, remote in-field measurements feasible. By employing low-power CMOS image sensors, near-infrared (NIR) LED light sources, and solar-powered electronics with GSM data transmission, it is now possible to develop a compact, watertight tomographic imaging system for long-term underwater deployment.

Here, we present a novel methodology applying optical tomographic imaging to a conventional sediment trap, enabling semi-continuous online monitoring of sediment accumulation. The specific objectives of this study were to (1) develop an autonomous, ultra-high-resolution tomographic sediment trap, (2) validate the volumetric measurement accuracy of the device in a controlled laboratory setting, and (3) deploy the prototype in a field environment (Savilahti Bay, Kuopio) to demonstrate its capability to monitor the short-term impact of onshore construction work on daily sediment yield. Ultimately, this methodology aims to provide crucial high-resolution data for tracking sediment dynamics, particularly in remote or hard-to-access study sites.

## Material and methods

### Site description

**Fig. 1:**
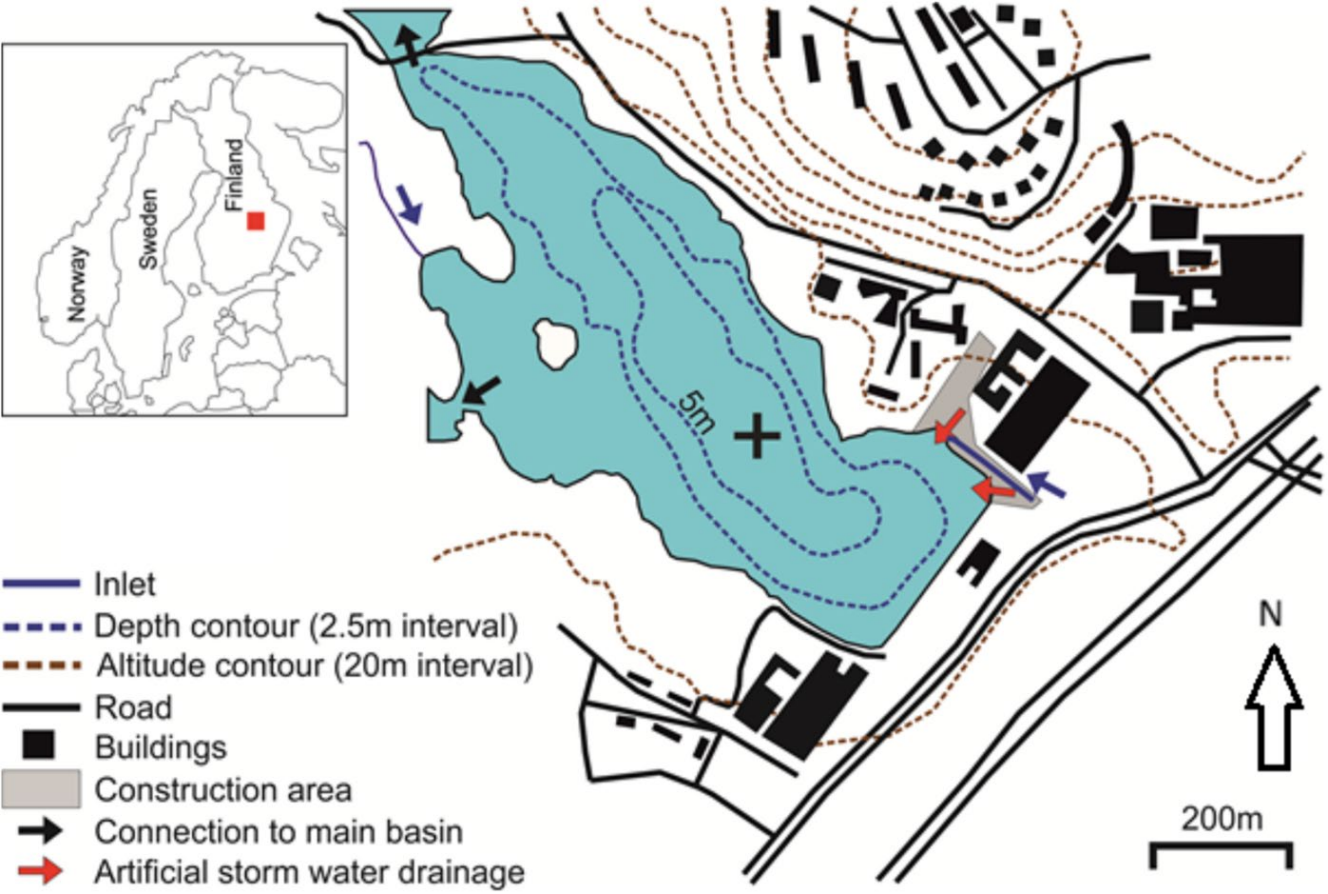
Location of Savilahti bay and the online sediment trap site in the deepest part of the basin (marked with +)

**Fig. 2:**
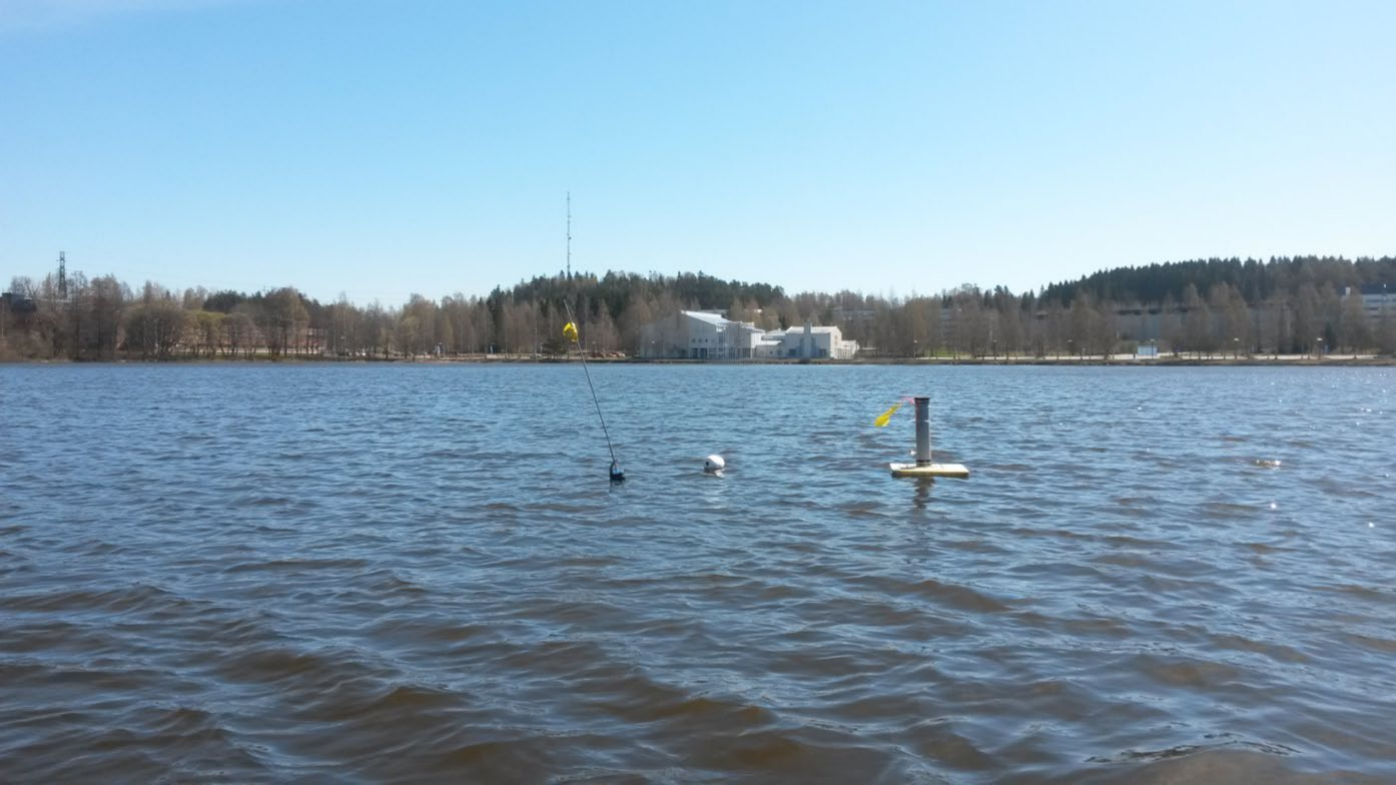
Online trap in Savilahti bay basin at a depth of 6 m under the flag sign. Anchored float behind flag sign is the base station for the online trap, which sends measurements to the Internet over a mobile network

The large water body of Lake Kallavesi surrounds the city of Kuopio, which has a population of 120 in eastern Finland. The complex Lake Kallavesi basin includes several semi-closed basins such as Savilahti Bay. Savilahti Bay was selected as the study site due to several advantages. With the University of Eastern Finland campus located on the shore, Savilahti bay was an ideal place to test the prototype of sediment trap system due to ease of access to the trap location in case the online data shows interruptions, disconnection, filling over maximum measuring volume or other problems. The surface area of the Savilahti basin is 0.345 km^2^, and maximum water depth is 7 m. There are two narrow streams discharging to the basin. The silled connections to the large Lake Kallavesi water body at the north and at eastern side of the Savilahti basin, enable surface water exchange between the two basins. A prototype of the high-resolution online trap was deployed in the deepest part of Savilahti Bay from October 22, 2017, to October 6, 2018 (Figs. 1 and 2). The trap was moored one meter above the lake floor. The construction work began at the instant shore of Savilahti Bay on 27th of July 2018 and continued until the end of the investigation period (Kuopion Energia, 2019). The detailed record of construction work and the possibility to start trap monitoring prior to construction work gives an ideal opportunity to measure daily changes in catchment erosion during the construction and evaluate the environmental risks related in detail.

**Fig. 3:**
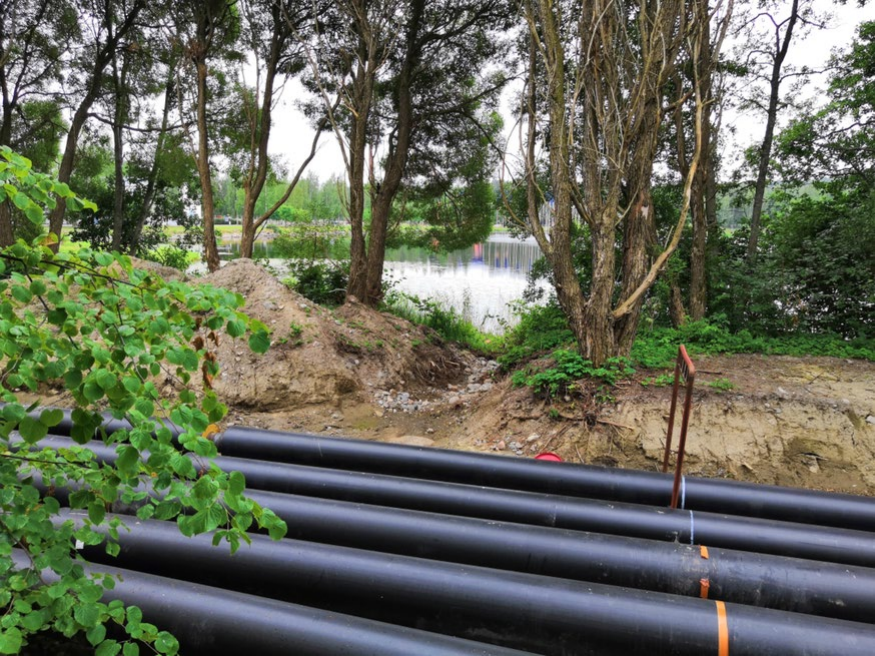
Construction in progress near the Savilahti bay, district heating and district cooling pipelines installed to a about 160 m long ditch. Photo taken from approximate coordinates 62°53′42.43″ N, 27°38′24.47″ E, midway along the trench

The construction site commenced with excavation for the installation of district cooling and heating pipes. The approximate starting and ending coordinates of the roughly 160-m-long open trench were 62°53′40.13″ N, 27°38′30.51″ E and 62°53′43.55″ N, 27°38′22.05″ E, respectively. The trench had an approximate cross-section of 1–1.5 m in depth and 3–5 m in width, and it was excavated in clay soil. The formation of several uncontrolled, rainwater-induced outwash channels from the excavation to the bay (Fig. 3) was observed in three different locations Suspended clay particles were transported from these channels into the bay via runoff. Concurrently, foundation structures for a central cooling plant were being constructed on an adjacent slope. The elevation difference between the construction area on the slope and the water level of the bay was estimated to be 20 m.

### Operating principle of the online trap

**Fig. 4:**
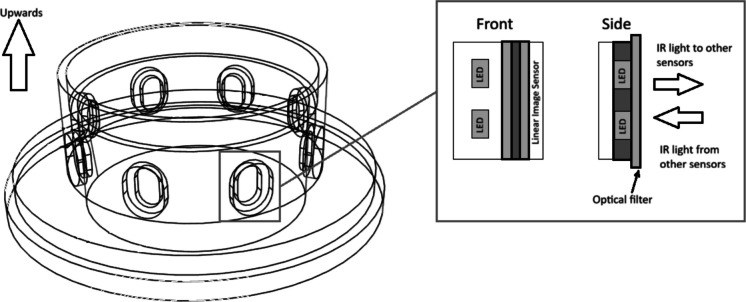
Technical drawing of the tomographic unit frame (derived from the CAD model). The detail view shows the arrangement of the NIR LEDs, the CMOS linear image sensor, and the optical band-pass filter

**Fig. 5:**
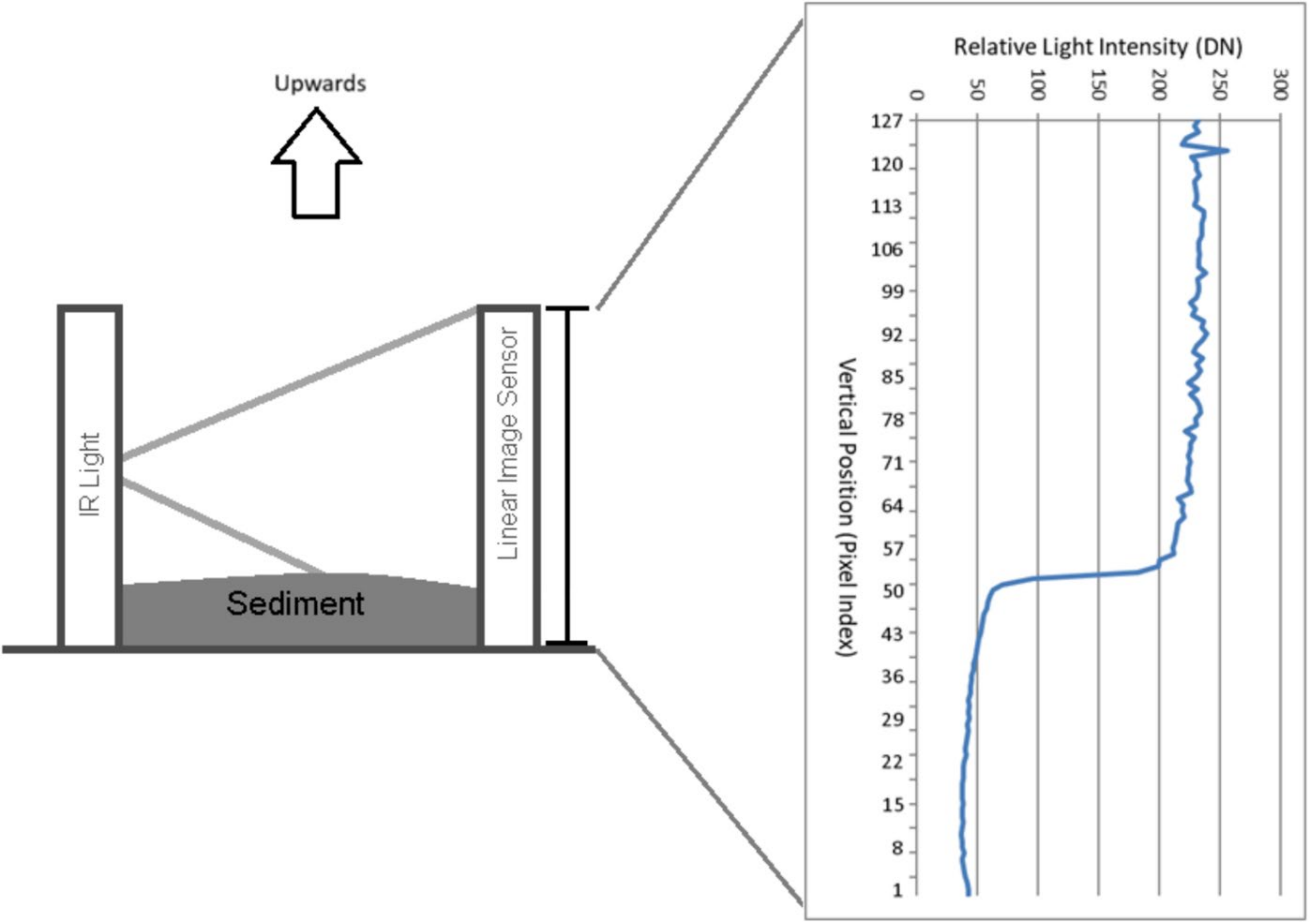
Operating principle of the optical measurement. (Left) Schematic representation of a single infrared (IR) transmitter–receiver pair, illustrating how the accumulated sediment attenuates the light path. (Right) An example of the light transfer profile, where the horizontal axis represents the relative light intensity (DN) (0 = total absorption, 255 = clear water). The vertical axis (0–127 pixels) indicates the spatial position, with the upward arrow signifying the direction of sediment accumulation from the bottom (0 mm) toward the top (8.125 mm) of the measurement area

**Fig. 6:**
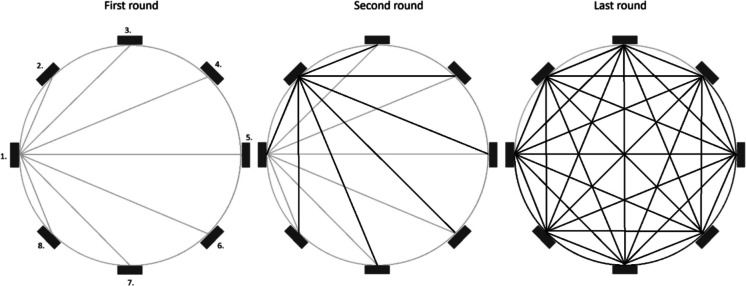
The sequential tomographic scanning process. The “First round” shows LED array 1 acting as a light source while cameras 2–8 act as receivers. The sequence follows Algorithm 1 until the “Last round” is completed. A complete cycle generates 56 independent vertical light paths for 3D reconstruction

The volumetric measurement of the online sediment trap is based on optical tomography using the near-infrared (NIR) spectrum. To eliminate interference from ambient solar radiation, the system utilizes NIR LEDs (880 ± 25 nm) paired with an 800–2200 nm band-pass filter. The structural design consists of a cylindrical frame housing eight vertically oriented CMOS line cameras and corresponding LED arrays positioned around the collector tube (inner diameter 56.5 mm) (Fig. 4). The system sequentially scans the tube, capturing multi-static 1D attenuation profiles to generate 56 independent vertical data planes per cycle (8 sources × 7 receivers) (Fig. 5 and Fig. 6). The measurement uncertainty of the system is ± 0.6 mL (determined through laboratory calibration), with a maximum measurable volume of 19.9 mL. The duration of a single measurement cycle is approximately 20 s. Detailed hardware specifications, specific component models, and the exact scanning algorithm are provided in the Supplementary Information.

### Measurement data processing and volume estimation

**Fig. 7:**
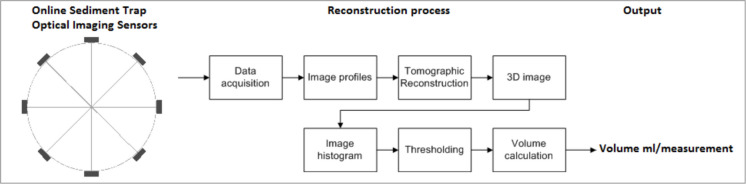
Tomography image process used in online trapping methodology

**Fig. 8:**
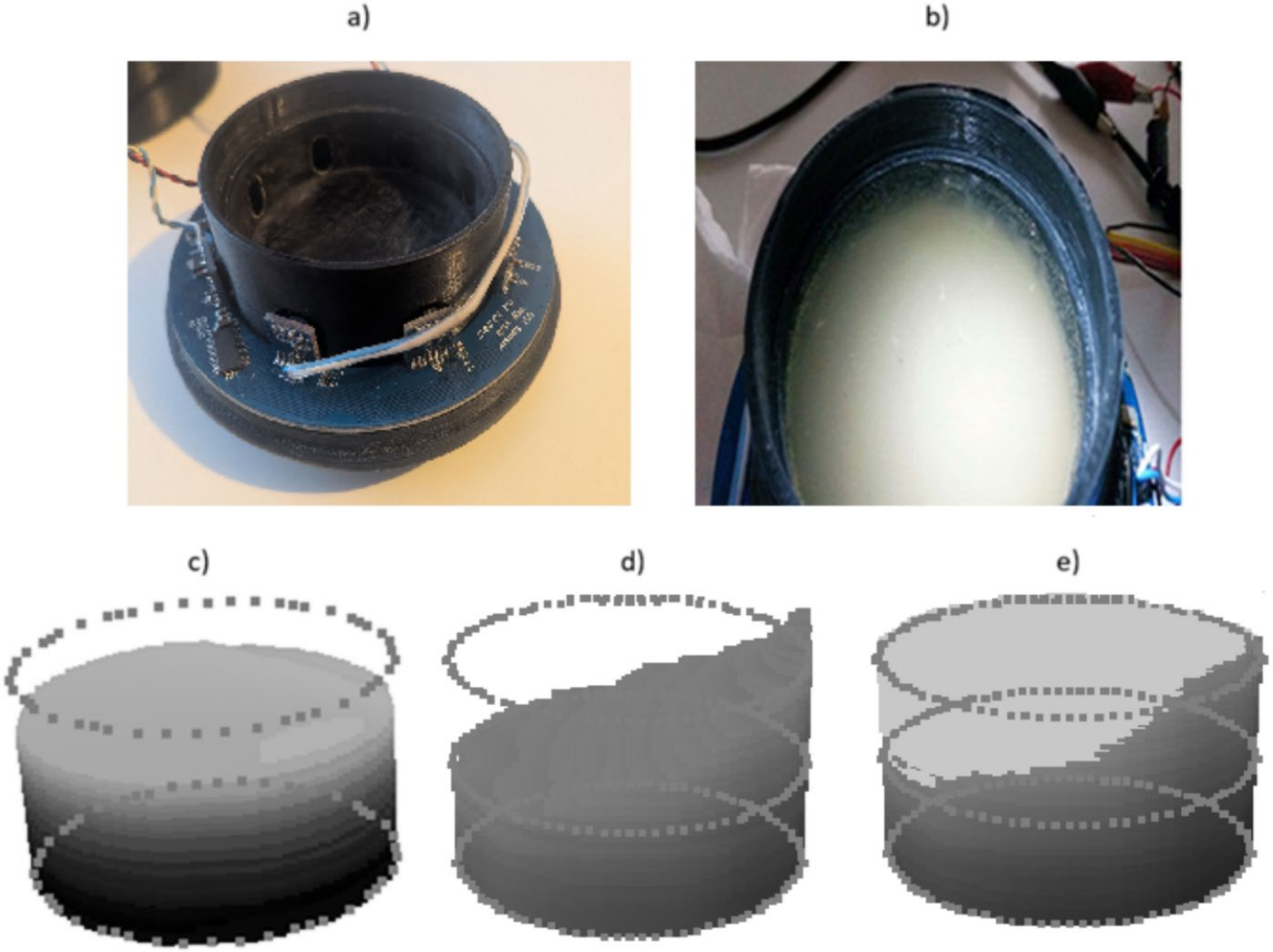
Technical design and tomographic reconstruction capabilities. (**a**) Internal structure showing the ring of optical sensors before encapsulation. (**b**) Laboratory setup for volumetric validation. (**c**) Tomographic reconstruction of a uniform sediment layer. (**d**) Demonstration of the system’s ability to reconstruct an asymmetrical/slanted sediment surface, confirming accurate volume integration regardless of surface geometry. (**e**) The impact of adaptive thresholding: the grayscale gradient is converted to a binary representation to exclude the water column from the volume calculation. This multi-path design and algorithmic processing provide a significant advantage over single-point measurement techniques by ensuring robust performance even in turbid conditions. Note: The circular outlines and lines in panels (**c**)–(**e**) are geometric overlays provided as visual aids to indicate the measurement chamber’s boundaries and the volumetric integration area; they do not represent physical internal structures

The captured light transfer profiles are processed using tomographic reconstruction to create a volumetric image of the trap’s interior. A Filtered Back-Projection (FBP) algorithm is used to reconstruct the data. In this process, the 1D projection profiles are first smoothed using a low-pass mean filter to reduce noise. These filtered profiles are then mathematically projected back (“smeared”) across a 2D plane from their respective measurement angles to form a cross-sectional slice of the sediment density (Fig. 7). By repeating this reconstruction for each vertical pixel level, the 2D cross-sections are stacked to form a complete 3D volumetric image. To distinguish the accumulated sediment from water and suspended particles, automatic image segmentation is performed using Otsu’s method (Otsu, 1979), which converts the grayscale reconstruction into a binary representation (Fig. 8e). The sediment volume is calculated by determining the cross-sectional area of the sediment segment at each level and integrating these areas over the entire measurement height (Fig. 8d). The detailed mathematical equations for the FBP reconstruction, Otsu’s thresholding, and volume integration are provided in Supplementary Information S1. Changes in the recorded sediment volume are categorized as either incremental or decremental. An increment represents active sediment deposition (flux), whereas a decrement reflects the natural compaction of the material already present in the trap. To determine the true sedimentation rate, only positive volume increments are added to the cumulative flux, while negative values update the baseline to account for continuous sediment consolidation over time.

### Field deployment and prototype design

**Fig. 9:**
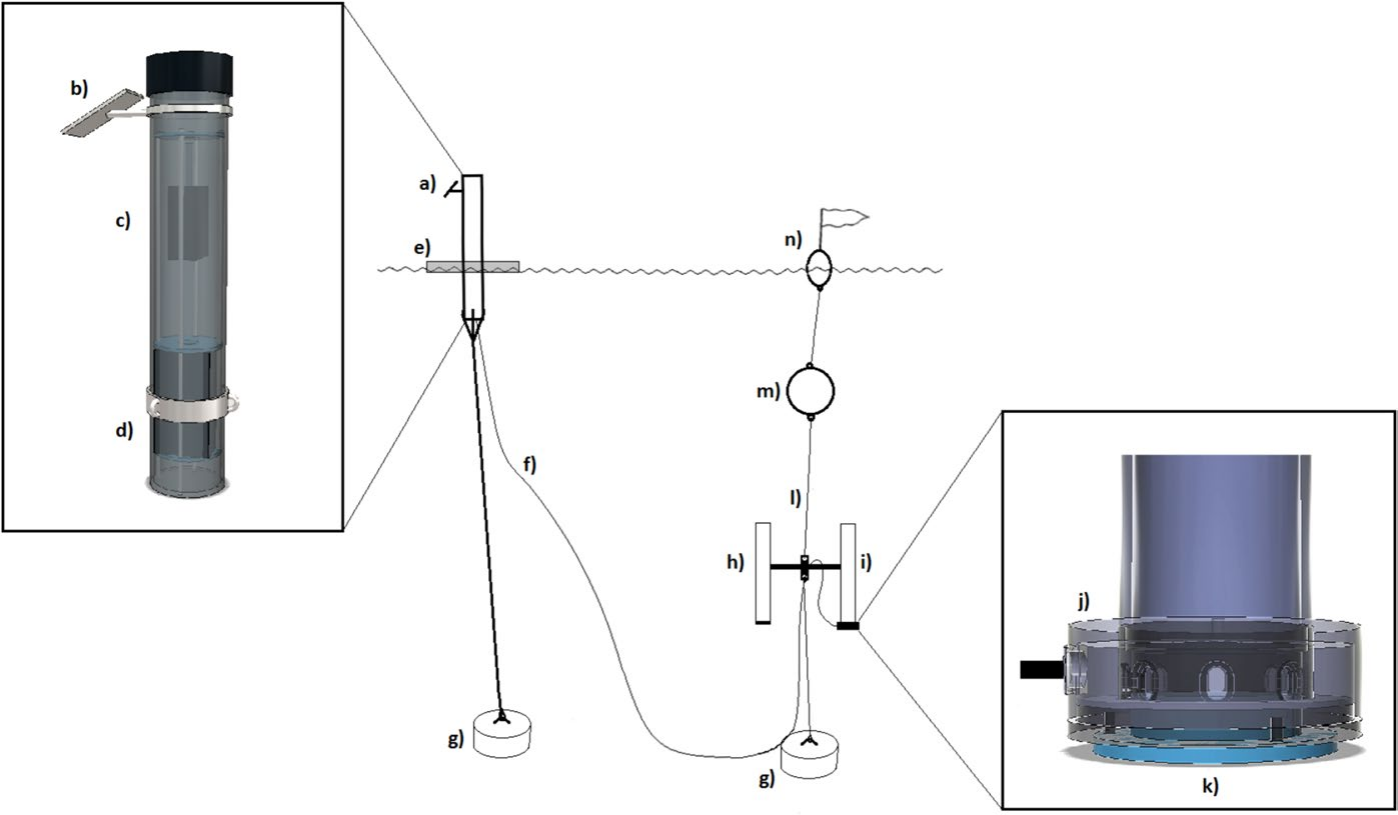
Online buoy system and trap assembly. (**a**) Online buoy, (**b**) solar panel, (**c**) data logger with cellular modem, (**d**) rechargeable battery, (**e**) raft, (**f**) cable connecting the tomographic imaging device to the online buoy, (**g**) anchor weight, (**h**) conventional collector tube, (**i**) collector tube with tomographic imaging device, (**j**) tomographic imaging device, (**k**) balancing weight, (**l**) sediment trap body, (**m**) float, and (**n**) flag sign buoy

A prototype of the online sediment trap was installed at the bottom of a conventional trap body. To validate the results, it was paired with a standard collector tube for manual volume comparison. Because establishing a direct underwater wireless data link is challenging, the imaging device was connected via cable to an anchored surface buoy (Fig. 9). The buoy housed a solar-powered data logger and a cellular modem, enabling fully autonomous operation. The system was designed for extreme energy efficiency, allowing it to record measurements hourly and transmit data daily without the need for battery replacements. Detailed electrical specifications, power consumption metrics, and data transmission protocols are provided in Supplementary Information S1.

### Volume calibration

Volume estimation is based on tomographic measurement and image processing, where the extracted sediment fraction is mathematically converted to milliliters. While the theoretical volume error is minimal, empirical measurement errors arising from optical component misalignment, numerical rounding, or nonlinear data processing must be determined to evaluate reliability. (Uneven sediment deposition caused by the tilting of the collector tube is unlikely, as an added bottom weight forces the tube into an upright position.) Volume calibration was performed by comparing known reference volumes to the volumes measured by the online imaging device. The calibration procedure consisted of a sequential series of clay suspension additions. First, a baseline measurement was taken from the collector tube filled only with distilled water (0 mL reference). Next, a 5 mL increment of clay was accurately pre-measured using a graduated cylinder and added to the tube to form a suspension (transmittance approximately 40%). The suspension was allowed to settle at the bottom of the tube for about 30 min until the water was clear again, after which the accumulated volume was measured by the tomographic device. This exact sequence adding a 5 mL clay increment, waiting 30 min for it to settle, and recording the tomographic measurement was repeated four times in total, until a cumulative known volume of 20 mL of clay was reached. This systematic procedure allowed for a precise assessment of the device’s measurement accuracy across its operational range.

### Data and observations

Data used in this study is based on online trap measurements with a time interval of 60 min and visual observations. The meteorological observations were obtained from open data of the Finnish Meteorological Institute services. Meteorological station is located on the shore about 200 m from the trap providing observations on air temperature (°C), wind speed (m/s) and water precipitation (mm). Ice cover information is based on visual observation from the Savilahti bay, that were recorded to a diary. All data, sediment accumulation and meteorological observations were combined to one dataset for statistical and time-series analysis. The ice cover data in the dataset were marked as 0 representing liquid water and 1 representing ice cover. Construction period data are based on visual observations. The construction data in the dataset were marked as 0 representing normal situation and 1 representing ongoing construction. All measurement data was collected during 22.10.2017 to 6.10.2018, where the normal period was during 22.10.2017–1.11.2017, 7.11.2017–1.12.2017 and 5.5.2018–26.7.2018. Dates, when the bay was covered by ice, were 2.11.2017–6.11.2017 and 2.12.2017–4.5.2018. Finally, the construction period was 27.7.2018–6.10.2018. Data were grouped to three groups, normal situation, ice cover and construction situations. Normal situation represents a group, where catchment erosion and sediment suspension mostly are caused by natural forcing factors, and ice cover group represents situations, where the bay was covered by ice. Final group, construction period, represents a situation where catchment erosion and sediment suspension mostly are increased by construction. Ice cover group was excluded from the statistical analysis.

### Statistical and time-series analysis

Statistical analysis was processed with SPSS software version 25.0 (IBM Corp, 2017). First, data were preprocessed by setting outlier values to variables and then calculating daily averages for continuous variables from one-hour resolution data. Discrete variables of water and snow precipitation were also preprocessed by setting outlier values to variables but calculating the daily sum instead of averaging. Data is visualized by plotting time-series diagram in one dimension, in order to inspect important features of time-series data including base level, peaks and changes. Construction and normal periods were divided into two categories using ice cover and construction information i) winter season, excluded from the dataset by ice cover information and ii) the rest of data. Construction and normal situations were grouped by construction information. To inspect the normal and construction periods in detail, the data were grouped to weekday groups (Monday to Sunday) with representation of averaged situations in each day. Construction work in the study site occurred only during weekdays. Period component in the time series was examined using autocorrelation function, which describes the correlation with different time lags of the time series to itself.

## Results and discussion

### Instrument validation and performance

**Fig. 10:**
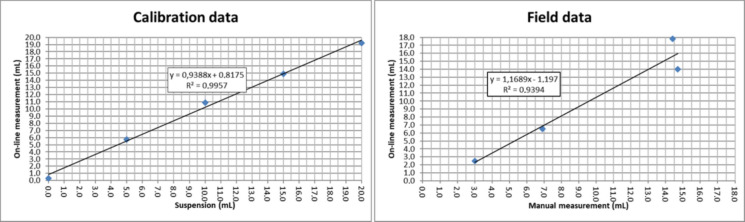
Volumes measured from artificial sediment additions (left) and field measurements from Savilahti (right)

The accuracy of the tomographic volume measurement was validated through laboratory calibrations and field experiments in Savilahti (Fig. 10). The laboratory calibration, covering a cumulative volume up to 20 mL, showed a slight systematic overestimation (less than 1 mL) at volumes between 0 and 10 mL. The correlation between the added reference volume and the tomographic measurement was excellent (*R*^2^ = 0.9954), supporting the previously estimated measurement uncertainty of ± 0.6 mL. Field validation was conducted by manually confirming the sediment volumes at four separate collection times during the prototype test year. The online measurements showed a strong correlation with the manually confirmed volumes (*R*^2^ = 0.9394), with a mean difference of 3.0%. Consistent with the laboratory results, the online measurements were slightly larger than the true volumes, except for the final sample where the online volume underestimated the manual measurement. This discrepancy occurs when the sediment trap fills close to its theoretical upper limit (19.9 mL). At this stage, the tomographic image histogram becomes nearly flat (variance near zero), preventing the Otsu thresholding algorithm from identifying a distinct boundary between sediment and water. This indicates that the practical maximum detection limit is slightly lower than the theoretical maximum, suggesting that image processing methods may require optimization for high-deposition areas or longer deployment periods.

**Fig. 11:**
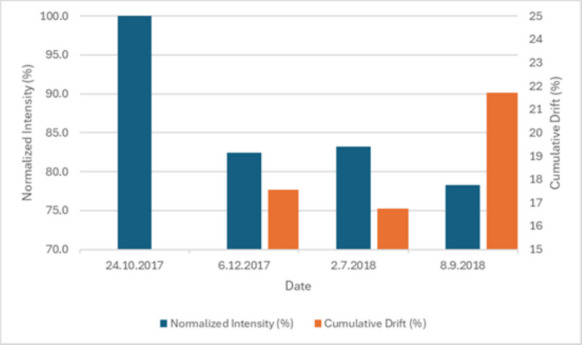
Long-term evaluation of optical sensor stability. The blue bars represent the normalized signal intensity (left axis), while the orange bars indicate the total signal attenuation relative to the initial calibration (right axis). After an initial stabilization phase, the attenuation remained remarkably consistent, confirming the long-term reliability of the measurement baseline despite the year-long underwater deployment

To further assess technical reliability, the long-term stability of the optical sensors was evaluated (Fig. 11). The normalized signal intensity of the reference sensors (unattenuated light paths) showed an initial adjustment period, stabilizing at 82.5% by December 2017. This 17.5% initial shift is attributed to the sensors settling into the underwater environment and the formation of a stable biofilm equilibrium. For the remainder of the 12-month deployment, the signal remained remarkably stable, with the change in total attenuation between December and September being only 4.9%. As the tomographic method is based on identifying the physical boundary between the settled sediment and the water column, it is inherently robust against signal attenuation. The adaptive thresholding algorithm recalibrates the “clear water” reference for each image, meaning the boundary detection remains accurate as long as sufficient optical contrast exists. This ensures that minor signal fluctuations or light biofouling do not compromise the volume determination. This long-term consistency confirms that the adaptive thresholding algorithm operated on a reliable baseline and that cumulative biofouling did not compromise the measurement accuracy even during extended submersion (Delauney et al., 2010). Sediment compaction was addressed in both settings. A 30-min consolidation period was observed in the laboratory before recording measurements. In the field, although compaction was detectable in high-resolution hourly data, its influence was minimized by averaging the measurements to a daily resolution, rendering the effect on calculated daily sedimentation rates insignificant. Furthermore, since the primary objective was to detect distinct high-deposition events rather than absolute density, the minor long-term compaction did not interfere with the event-based analysis or the overall flux patterns.

### Case study: sedimentation in Savilahti Bay

**Fig. 12:**
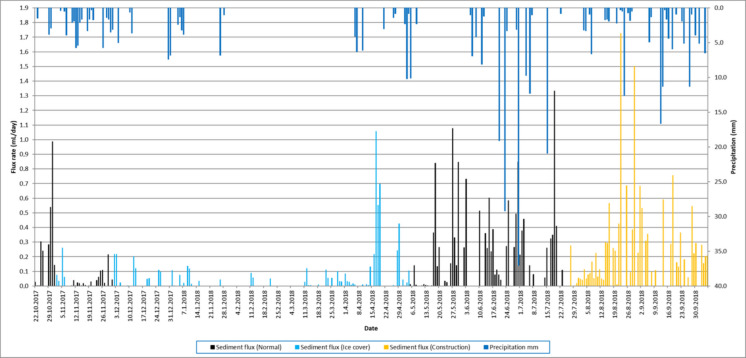
Daily sedimentation rate and precipitation in Savilahti bay from October 22, 2017, to October 6, 2018. The columns indicate the sediment flux (mL/day), with colors representing different environmental conditions: sediment flux under normal (black), ice cover (light blue), and construction work (orange). The dark blue bars represent daily precipitation (mm). Seasonal variations and episodic flux peaks are clearly visible across the different monitoring periods

**Fig. 13:**
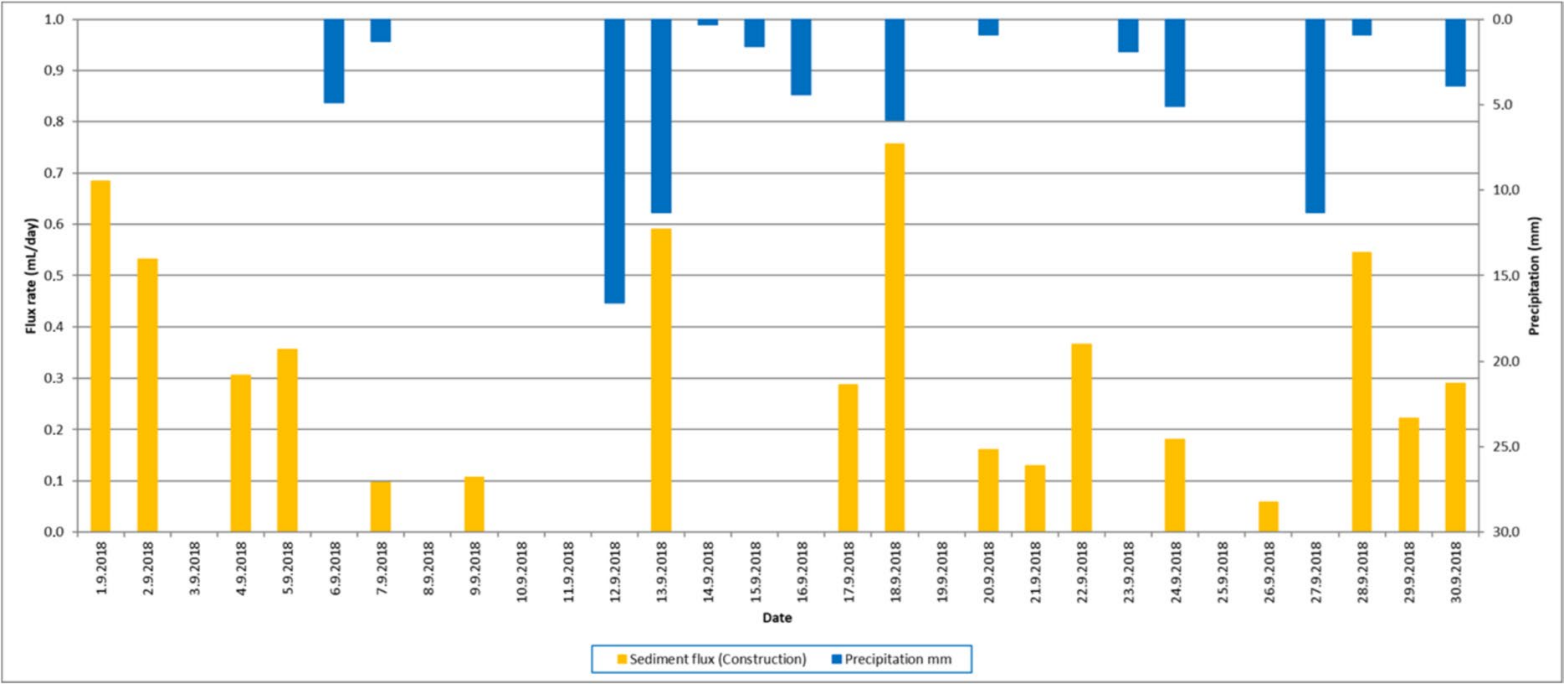
Detailed view of the event-based response of the sediment trap in September 2018. The orange columns indicate the daily sediment flux (mL/day) during the construction period, and the dark blue bars represent daily precipitation (mm). A significant peak in sediment transport is observed immediately following the heavy rainfall event on September 12–13 (totaling 28.5 mm), demonstrating the high temporal resolution and sensitivity of the tomographic measurement

**Fig. 14:**
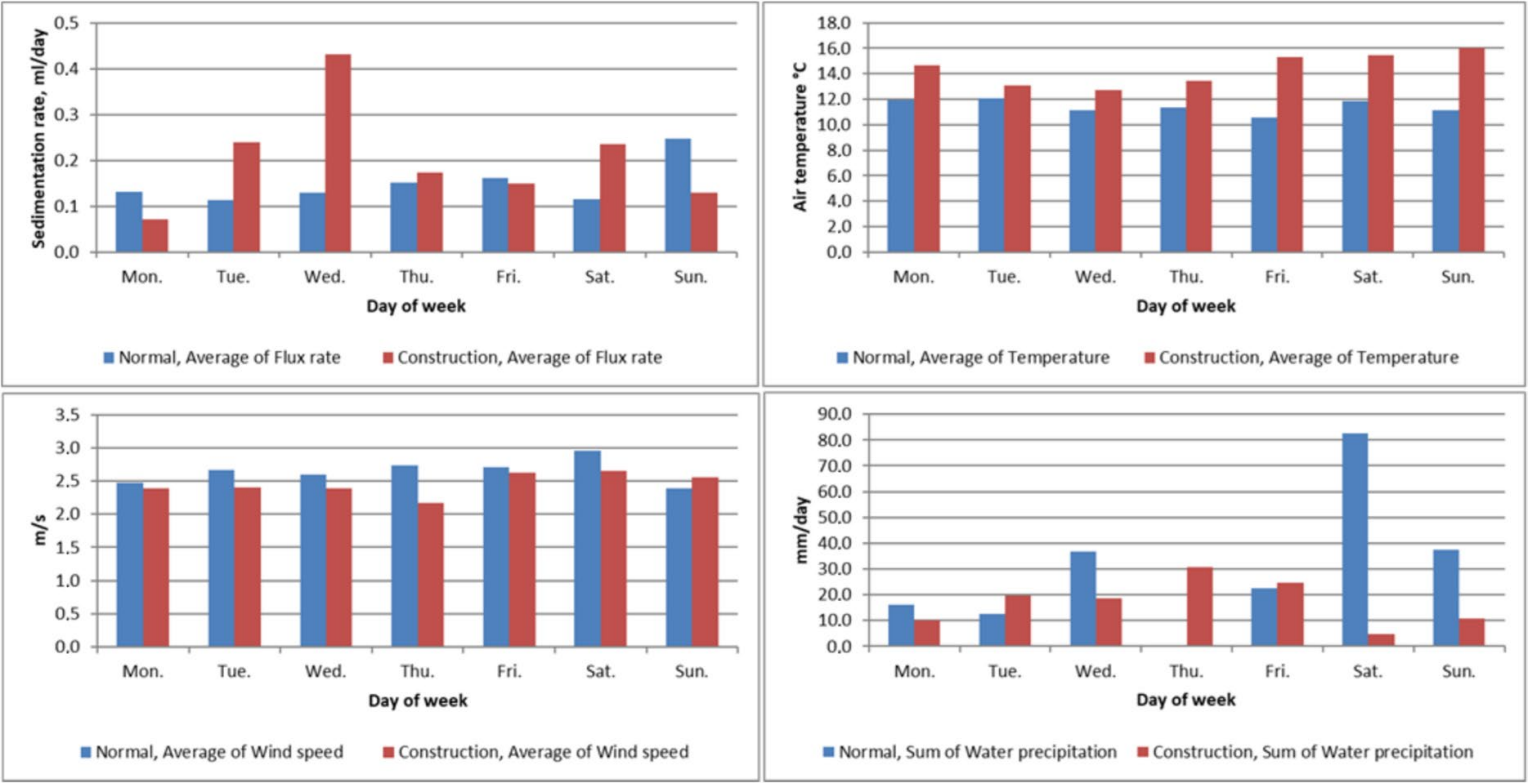
Normal (blue bar) and construction (red bar) situations grouped by weekday. (**a**) Average flux rate of the group. (**b**) Average wind speed of the group. (**c**) Average temperature of the group. (**d**) Average water precipitation of the group

The volume measurement time series show large seasonal and daily variations in sedimentation rates (Fig. 12). Seasonal conditions control the availability of sedimentary material (Johansson et al., 2019; Saarni et al., 2017; Zolitschka et al., 2015), and sediment accumulation is considerably smaller during the winter season when ground frost and ice cover prevent material transport from the catchment to the lake. During this period, organic production is at its minimum, and several days with a sedimentation rate of 0 mL/day occur. The maximum accumulation rate of 1.73 mL/day occurred on August 22, 2018. Data analysis comparing daily sedimentation rates against meteorological data series shows that the sediment accumulation increases as a consequence of precipitation events (Johansson et al., 2019). Vertical temperature profiles were monitored from the surface to the bottom at 1 m intervals to characterize the environmental conditions during the deployment. Until July 26, 2018, the data showed a typical dimictic stratification pattern. While surface temperatures peaked during the summer months, the bottom water at the depth of the sediment trap remained consistently cooler, ranging between 4 °C and 8 °C for the majority of the spring and early summer period. Consistent with these observations, the volume of accumulated sediment was generally higher in relation to warmer surface temperatures and ice-free periods, suggesting the importance of frost- and ice-free conditions on sedimentation. We compared the sedimentation rate variations during the growing season—from the date when the ground was frost- and snow-free and the lake ice-free (May 1, 2018)—until the end of the measuring period. The data series were observed prior to construction work (May 1–July 26, 2018) and during the construction work (July 27–October 6, 2018) to measure changes in the sedimentation rate caused by active construction at the lake catchment and shoreline. There was no significant difference between the mean air temperature, wind speed, or precipitation between the pre-construction and construction periods. However, several individual heavy precipitation events occurred during the pre-construction period that may have significantly increased the sedimentation. Even so, the pre-construction sediment flux varied from 0 to 1.33 mL/day with a mean sedimentation rate of 0.15 mL/day, while the variation during the construction period was between 0 and 1.73 mL/day with a mean flux rate of 0.20 mL/day (Table 1). The high-resolution temporal data revealed a clear connection between weather events and sediment transport. For example, a significant precipitation event between September 12 and 13, 2018 (totaling 28.5 mm), was immediately followed by a distinct peak in the measured sediment accumulation (Fig. 13). In general, data shows a lag/delay of approximately 0–2 days between the precipitation event and peak sediment accumulation (Figs. 12 and 13). This demonstrates the trap’s ability to record rapid environmental responses that would be lost in traditional long-term cumulative sampling. Further analysis of the sedimentation rates during the construction period, grouped by weekdays, shows the cumulative impact of anthropogenic activity alongside these environmental drivers (Fig. 14).

**Table 1:** Descriptive statistics of variables on total dataset (top), normal (middle) and construction (bottom) periods

Over the year (22.10.2017–6.10.2018)						
Variable	Unit	*N* (days)	Minimum	Maximum	Mean	Std. deviation
Flux rate	mL/day	350	0.00	1.73	0.11	0.23
Air temperature	°C	350	−21.10	25.37	5.02	10.87
Wind speed	m/s	350	0.88	5.83	2.49	0.90
Water precipitation	mm/day	350	0.00	37.00	1.11	3.58
Normal period (22.10.2017–1.11.2017, 7.11.2017–1.12.2017, 5.5.2018–26.7.2018)						
Variable	Unit	*N* (days)	Minimum	Maximum	Mean	Std. deviation
Flux rate	mL/day	118	0.00	1.33	0.15	0.25
Air temperature	°C	118	−4.75	25.37	11.45	8.24
Wind speed	m/s	118	0.99	5.83	2.64	1.01
Water precipitation	mm/day	118	0.00	37.00	1.75	5.28
Construction period (27.7.2018–6.10.2018)						
Variable	Unit	*N* (days)	Minimum	Maximum	Mean	Std. deviation
Flux rate	mL/day	71	0.00	1.73	0.20	0.31
Air temperature	°C	71	1.03	24.88	14.41	5.69
Wind speed	m/s	71	0.99	4.70	2.46	0.76
Water precipitation	mm/day	71	0.00	16.60	1.66	3.30

To evaluate the impact of the construction work on sediment accumulation, the daily flux rates of the defined “normal period” (*N* = 118 days) were compared to the “construction period” (*N* = 71 days). A two-sample Welch’s *t*-test indicated that although the mean sediment flux rate during the construction period (mean = 0.20 mL/day) was higher than during the normal background period (mean = 0.15 mL/day), this increase was not statistically significant at the 95% confidence level (*p *= 0.21). This suggests that while there is an observable quantitative shift in the average accumulation, the high natural variance across different seasons (including spring meltwaters) limits definitive statistical confirmation. Nevertheless, the descriptive statistics align with the observation of an elevated sediment load entering the basin during the active earthworks.

Comparison of normal and construction periods by weekday grouped data (Fig. 14) shows a clear difference between the two periods. Sedimentation flux rate rises during weekdays compared to weekend days. The higher accumulation on Saturdays and very low accumulation rates on Mondays suggest one-day lag/delay between the event causing the erosion/sediment loading and the detected sedimentation at the bottom of the basin, which is in line with our observations related to erosion and sediment load related to precipitation. This likely results from the time required for transport and vertical sinking of the particles through the water column. On construction period, average of flux rate is higher on Tuesdays and Wednesdays, than normal construction situations.

**Fig. 15:**
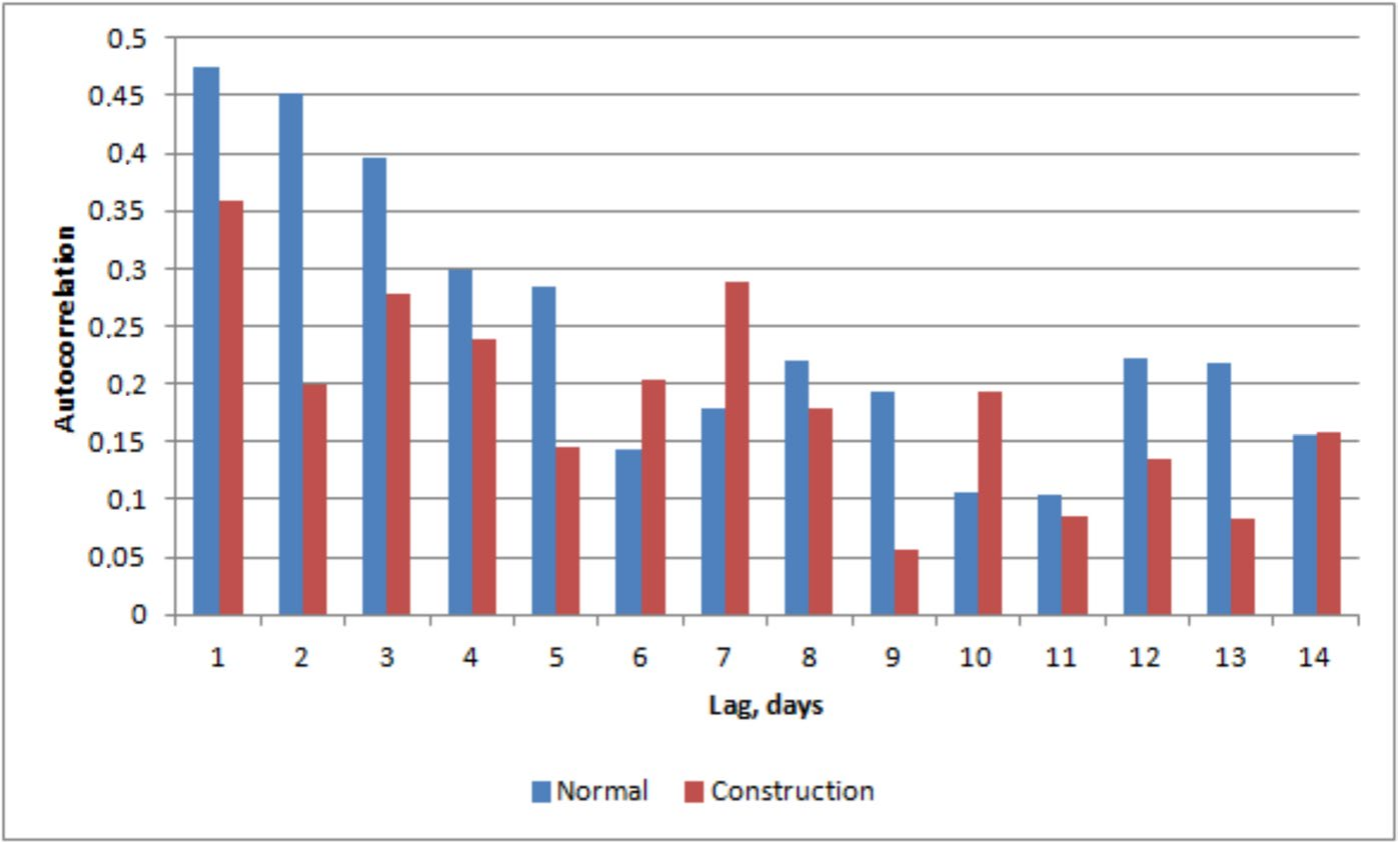
Autocorrelation of sedimentation flux rate on normal (blue bar) and construction (red bar) situations

Determining periodic components for normal and construction situations using autocorrelation (Fig. 15) reveals a distinct difference between the two periods. In normal conditions, the autocorrelation indicates a lack of repeated patterns or periodic components. In contrast, the construction period shows clear periodicity with 3- and 7-day cycles. These results suggest that construction work in the catchment enhanced sediment flux by 33%, despite the fact that major precipitation events during the normal period would have typically suggested higher erosion rates. The decrease in sediment flux during weekends, when construction was halted, and the rapid increase from Mondays to Wednesdays suggest a quick catchment response to construction activities with only a 1-day lag. These periodic components further indicate that construction work significantly influences catchment erosion and sediment suspension. A clear period of significantly increased sediment flux was observed from 14 August to 5 September (Fig. 12), reaching a peak of 1.73 mL/day. This coincided with the excavation and foundation work for a central cooling plant at the hillside construction site, where artificial storm water drainages led directly to the Savilahti bay.

Normal conditions, without repeating patterns indicating that sediment flux-rate occurs randomly, while during construction period a repeating pattern in sediment flux-rate occurs. This indicates sediment flux-rate increases induced by construction work.

### Technical validation and device reliability

The operational success of the sediment trap in the variable conditions of Savilahti bay can be attributed to its specialized structural and optical design that acknowledges issues related to biofouling, turbidity, and pressure resistance. A common challenge for underwater optical sensors is biofouling. In our device, the electronics and optical components are encapsulated within a protective plastic housing filled with epoxy resin. The optical path is embedded in clear epoxy, while the rest is sealed in opaque black epoxy. This solid-state construction eliminates internal air gaps and complex seals. While bacterial biofilms can form on any submerged surface, several factors minimized their impact. Vertical temperature profiles showed that the bottom water remained consistently cold (4–8 °C) during the primary deployment phase. This thermal stability, combined with the benthic darkness, suppressed microbial metabolic rates. Furthermore, the flush-mounted, vertical orientation of the optical surfaces prevented the gravitational settling of debris. As demonstrated in the performance validation, the signal drift remained minimal (4.9%) throughout the year-long study. In more productive or warmer environments where biofouling is more aggressive, the system can be enhanced with active or passive mitigation strategies, such as the integration of UV-C LEDs to deliver brief germicidal pulses onto the optical windows, or the use of copper-alloy bezels. However, our practical experience from a variety of trap applications in boreal waters (Saarni et al., 2023; Salmela et al., 2022) shows that even in near-surface environments with strong algal growth outside the trap, the interior typically remains free from growth. This is likely due to restricted water mixing, leading to a rapid decline in oxygen and nutrients inside the tube. To address concerns regarding water turbidity, the device was designed with a minimal optical path length (less than 10 cm). This short distance ensures that light scattering remains negligible even during high-turbidity events, such as the heavy runoff recorded in September 2018. The contrast remained sufficient for the image analysis algorithms to provide accurate volume measurements throughout the monitoring period. Finally, the solid-block epoxy construction provides exceptional resistance to hydrostatic pressure. Unlike traditional hollow-body instruments that rely on O-rings and thick-walled housings to prevent collapse, our encapsulated design is fundamentally less vulnerable to structural failure or leaks. While the current prototype was tested in the relatively shallow waters of Savilahti, the “solid-state” principle allows for potential deployment in significantly deeper lacustrine or even marine environments without major structural modifications.

### Environmental interpretation of high-resolution sediment flux

The high temporal resolution of the online trap enabled a detailed examination of the sediment flux in relation to local events. In Savilahti Bay, the most prominent feature in the data was the clear difference between weekdays and weekends during the construction period. The sediment flux was consistently higher during the working hours of the onshore earthworks, while it dropped to near-baseline levels during the weekends when the construction activity ceased. This immediate response suggests that the onshore activity had a direct and rapid impact on the sediment load entering the aquatic system. Furthermore, the high-resolution data allowed us to observe the interaction between precipitation events and construction-related erosion. For instance, the heavy rainfall recorded in late August and September led to significant sediment pulses, but these pulses were notably larger during the active construction phase than during similar rain events in the normal background period. This indicates that the exposed soil at the construction site was highly vulnerable to surface runoff, leading to an amplified sediment yield. Traditional sediment traps, which are typically emptied once a month or once a season, would have averaged these pulses over a long period, losing the information about the exact timing and the direct link to the construction work. Our methodology provides the necessary temporal detail to distinguish between natural background flux and anthropogenic impacts, which is crucial for effective environmental monitoring and the enforcement of water protection regulations.

### Limitations and future perspectives

While the presented tomographic sediment trap performed reliably in the boreal lake environment, certain physical and environmental limitations must be acknowledged. The optical measurement principle is sensitive to extreme water turbidity; if the concentration of suspended solids exceeds the sensor’s dynamic range, the NIR signal may be fully attenuated, leading to increased measurement uncertainty. Additionally, the current prototype is designed for shallow to medium depths (up to 20 m). Future development of the device could include the integration of automated anti-fouling mechanisms, such as mechanical wipers or UV-C LEDs, to prevent biofilm growth on the optical windows during long-term deployments in high-productivity estuarine or marine waters. Furthermore, the high temporal resolution of the system provides a robust platform for future studies integrating machine learning algorithms to automatically classify sediment types based on their optical properties in real-time. This technology aligns with the objectives of environmental monitoring frameworks, such as the EU Water Framework Directive, by providing the continuous data needed for proactive water quality management.

## Conclusions

There is a growing need for modern instrumentation in conventional sediment trapping to update environmental monitoring. The tomography method coupled with online technology enables sediment flux measurements with even hourly resolution without increasing monitoring efforts or costs. This study demonstrated that the solid-state design, utilizing epoxy encapsulation, provides a robust and pressure-resistant solution for long-term monitoring in diverse lake environments. The ultra-high-resolution sediment accumulation rate time series acquired using online sediment trapping provides valuable information about the response of a catchment to weather conditions and anthropogenic land use. The sediment flux variations with hourly resolution enable a better understanding of the instant catchment response to individual weather events or to specific land use activities and construction phases. Specifically, the high sensitivity of the method allowed for the identification of immediate flux peaks following heavy precipitation and the differentiation between natural background variation and anthropogenic drivers. Such knowledge can facilitate the selection of the most convenient protection, timing and conservation methods for a specific site and enables more precise risk assessments. Daily sedimentation rate time series help to target a specific source of enhanced erosion and sediment transport, and hence allow a better understanding of the influence of single meteorological events such as heavy rainfall or increased wind speed that are often crucial aspects in reconstructing past climate using sediment records. Utilizing conventional sediment trapping with deployment periods of weeks makes it difficult to address short-term events or single anthropogenic activities in detail. The ultra-high-resolution sediment flux time series sheds light on catchment responses to anthropogenic land use changes but can also be used to investigate the impact of seasonal conditions and single meteorological events. This allows for monitoring the impact of storm waters, flood peaks, and construction work. The online trapping technique seems to be a very powerful instrument for sediment flux studies to help in directing efforts to the sediment sources in space and time. The absence of significant biofouling or signal drift during the year-long deployment confirms the reliability of the optical tomographic approach for long-term environmental assessment. The identification of the influence of specific actions on the catchments of freshwater systems would facilitate conservation and protection during and after construction projects. Continuous, unnaturally strong sediment flux could potentially harm benthic animal communities. However, by using online monitoring, it would be possible to keep sediment flux at a sustainable level.

## Supplementary Information

Below is the link to the electronic supplementary material.

ESM 1 (DOCX 21.2 KB)

## Data Availability

The datasets generated during and/or analyzed during the current study are available from the corresponding author on reasonable request.
