# Supplementary material for: An online tomographic sediment trap for high-resolution environmental monitoring
Source: Environ Monit Assess. 2026 May 22;198(6):638. doi: 10.1007/s10661-026-15469-w (PMC13197281; doi:10.1007/s10661-026-15469-w)
Supplement: Supplementary file 1 — (DOCX 21.2 KB) [file 10661_2026_15469_MOESM1_ESM.docx]

Supplementary Information S1

1. Hardware specifications

- NIR LEDs with a peak emission at 880 ± 25 nm (Model XZTHI54W, Manufacturer SunLED)
- CMOS line cameras 128x1 pixels, 63.5 µm pixel pitch (Model TSL1401CL, Manufacturer ams-OSRAM AG)

2. Algorithm 1

Tomographic Scanning Cycle

| **FOR** each LED source i from 1 to 8:  Activate LED array i  **FOR** each CMOS camera j from 1 to 8:  **IF** j ≠ i:  Capture 1D vertical profile (128 pixels) from camera j  Store profile as projection Pi,j  **END IF**  **END FOR**  Deactivate LED array i  **END FOR** |
| --- |

In each cycle, the process initiates with the "First round" (LED 1 active), continuing through subsequent stages until the "Last round" (LED 8 active) is completed. Because each CMOS unit is a line sensor, a single capture records the entire vertical profile (pixels 0–127) simultaneously. This results in a multi-static measurement configuration where each scan cycle produces 8 × 7 = 56 independent vertical data planes. These projections (*P_i,j_*) are processed using a back-projection algorithm to reconstruct the cross-sectional "slice" of the sediment density. By repeating this process across the vertical range of the line cameras, a full three-dimensional representation of the accumulation is formed. This tomographic approach allows for accurate volume estimation even when the sediment surface is uneven or tilted. The measurement range in the vertical direction is 0 to 8.125 mm. With a collector tube inner diameter of 56.5 mm, this provides a maximum measurable sediment volume of approximately 19.9 mL. The measurement uncertainty of the system is estimated at ±0.6 mL, determined through laboratory calibration with known sediment volumes. The scan interval is programmatically adjustable, and the total duration of a single measurement cycle is approximately 20 seconds.

**3. 3D Reconstruction and Volume Estimation**

The three-dimensional reconstruction of the sediment volume is achieved by processing the captured projections as a stack of independent horizontal cross-sections along the vertical Z-axis. Each vertical pixel level *k* (where $k\in[0,Z_{HEIGHT}-1]$) and the total number of layers is *Z_HEIGHT_* = 128. For each level *k*, a 2D tomographic reconstruction *f(x, y, k)* is calculated using a discrete implementation of the Filtered Back-Projection (FBP) algorithm

$$f\left( x,y,k \right)=\sum_{i=1}^{8} {Q_{\theta}}_{i}\left( x\cos\theta_{i}+y\sin\theta_{i},k \right)\Delta\theta$$

Where:

- *k* is the vertical pixel index on the Z-axis, representing a specific height level.
- ${Q_{\theta}}_{i}$ is the filtered projection data at height *k* for the *i*-th LED source
- $\theta_{i}$ is the projection angle of each LED array.

The Filtered Back-Projection (FBP) algorithm is a standard analytical method used to reconstruct a 2D image from its 1D projections. In a simple back-projection, the 1D profiles are projected back onto the image grid at their original acquisition angles. However, this basic approach results in a blurred "star artifact" around high-density areas due to the accumulation of low-frequency data in the center of the reconstruction. To compensate for this, the FBP algorithm introduces a filtering step before the back-projection. Each 1D projection is convolved with a high-pass filter specifically a Ram-Lak (Ramp) filter which suppresses low-frequency components and enhances sharp edges. In our implementation, the reconstruction is performed on a 56 x 56 pixel grid, matching the horizontal measurement geometry. Each level represents a 63.5 µm thick horizontal slice along the Z-axis. When these filtered profiles are subsequently back-projected and summed, the blurring is cancelled out, resulting in a sharp and mathematically accurate representation of the original cross-section. The full 3D volume is formed by stacking these *Z_HEIGHT_* horizontal slices. The final reconstructed 3D volume consists of a 128 x 56 x 56 voxel matrix (where *Z_HEIGHT_* = 128 and diameter = 56), representing a total of 401 408 independent volumetric elements. Given the vertical resolution of 63.5 µm and the horizontal grid scaled to the 56.5 mm collector tube, each voxel provides a high-density spatial representation. This resolution is sufficient to capture subtle changes in sediment surface topography and internal consolidation processes over time.

To distinguish the accumulated sediment from the surrounding water, Otsu’s method (Otsu, 1979) for automatic threshold selection is employed. This algorithm calculates an optimal threshold *t* by minimizing the intra-class variance $\sigma_{w}^{2}(t)$, which is defined as a weighted sum of variances of the two classes (sediment and water):

$$\sigma_{w}^{2}\left( t \right)=w_{0}\left( t \right)\sigma_{0}^{2}\left( t \right)+w_{1}(t)\sigma_{1}^{2}(t)$$

Where:

- *w_0_* and *w_1_* are the probabilities of the two classes separated by threshold *t*, and $\sigma_{0}^{2}(t)$, and $\sigma_{1}^{2}(t)$, are their respective variances.

After thresholding, the cross-sectional area of the sediment *A_k_* is calculated for each level $k$. Finally, the total accumulated sediment volume *V* is determined by integrating (summing) these areas over the entire measurement height:

$$V=\sum_{k=0}^{127} A_{k}\cdot\Delta_{z}$$

Where:

- $\Delta_{z}$ represents the vertical resolution of the system (63.5 µm per pixel), defining the thickness of a single *k*-level on the Z-axis.

**4. Prototype electrical and telemetry specifications**

Communication between the buoy and the imaging device utilized serial communication via RS-485 transceivers at a transfer rate of 115,200 bps. The cable also served as the power line for the imaging device. The buoy housed a 12 V lead-acid battery with a 3.2 Ah capacity. The battery was positioned at the bottom of the buoy, below the waterline, to maintain stable temperature conditions and act as ballast for stability. A 18 V / 3 W solar panel was used to recharge the battery during daylight hours. The total average current consumption for the electronics in both the buoy and the imaging device was approximately 650 µA. The peak current during measurement was approximately 100 mA, increasing to 2 A during mobile network transmission. With solar charging, the estimated operating time exceeds one year, and approximately 160 days without solar input. Data was stored hourly on a Secure Digital (SD) card and transmitted daily at 23:00 via email using the SMTP protocol (Simple Mail Transfer Protocol). If the transmission was successful, the SD card was cleared; otherwise, data was retained until the next successful transfer.
